# Supplementary material for: Glycan-induced structural activation softens the human papillomavirus capsid for entry through reduction of intercapsomere flexibility
Source: Nat Commun. 2024 Nov 21;15:10076. doi: 10.1038/s41467-024-54373-0 (PMC11582657; doi:10.1038/s41467-024-54373-0)
Supplement: Supplementary file 1 — Supplementary Information [file 41467_2024_54373_MOESM1_ESM.pdf]

Supplementary Information

Glycan-induced structural activation softens the human papillomavirus capsid for entry through reduction of intercapsomere flexibility

Yuzhen Feng[1#], Dominik van Bodegraven[2#], Alan Kádek[3,4#], Ignacio L.B. Munguira[1], Laura Soria-Martinez[2], Sarah Nentwich[3], Sreedeepa Saha[2], Florian Chardon[2], Daniel Kavan[4], Charlotte Uetrecht[3,5*], Mario Schelhaas[2*], Wouter H. Roos[1*]

[1]Moleculaire Biofysica, Zernike Instituut, Rijksuniversiteit Groningen, Groningen, Netherlands

[2]Institute of Cellular Virology, ZMBE, University of Münster, Münster 48149, Germany

[3]CSSB Centre for Structural Systems Biology, Deutsches Elektronen-Synchrotron DESY & Leibniz Institute of Virology (LIV), Notkestraße 85, 22607 Hamburg, Germany

[4] Institute of Microbiology of the Czech Academy of Sciences, Videnska 1083, 14220 Prague, Czech Republic

[5]Institute of Chemistry and Metabolomics, University of Lübeck, Ratzeburger Allee 160, 23562 Lübeck, Germany

[#]these authors contributed equally

*corresponding authors:

charlotte.uetrecht@cssb-hamburg.de for HDX-MS related queries,

schelhaa@uni-muenster.de for HPV related queries,

w.h.roos@rug.nl for AFM related queries

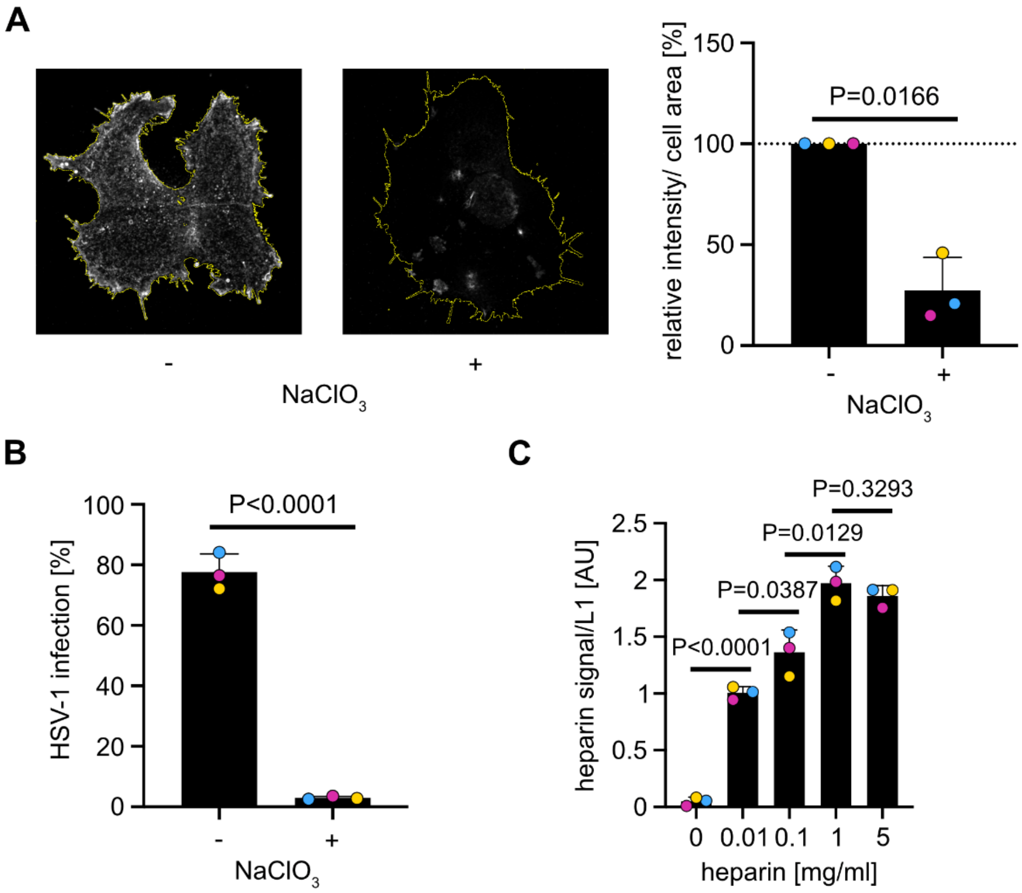

Figure S1: Consequences of sodium chlorate ($NaClO_3$) treatment on cellular HS and concentration-dependent heparin engagement of HPV16 PsV

(A) Immunofluorescence analysis of HaCaT cells with and without $NaClO_3$ treatment using an antibody directed primarily against sulphated HS. The samples were analysed by confocal microscopy 48h post $NaClO_3$ treatment. Single cells are displayed as maximum intensity projections of confocal stacks (left). Cell outlines are derived from phalloidin staining. Quantification to the right: At least 10 fields of view per independent experiment were analysed computationally to measure fluorescence intensity of sulphated HS staining. Quantified signals per cell area were displayed as average in % relative to untreated cells for three independent experiments ± SD. (B) Infection with HSV-1 expressing GFP was used to infect HaCaT cells treated with $NaClO_3$ or untreated. GFP expression was detected 6 h p.i. by flow cytometry. Infected cells were displayed as average in % of total cells relative to infection of untreated cells for three independent experiments ± SD. (C) HPV16 PsV were incubated with increasing amounts of biotinylated heparin as indicated for 1h, separated from unbound heparin by ultracentrifugation through an optiprep step gradient, and heparin was quantified by ELISA after incubation with streptavidin-conjugated HRP. Depicted are HRP signal intensities (heparin quantity) per L1 amounts (mean of three independent experiments) ±SD. AU represents arbitrary units. Source data are provided as a Source Data file.

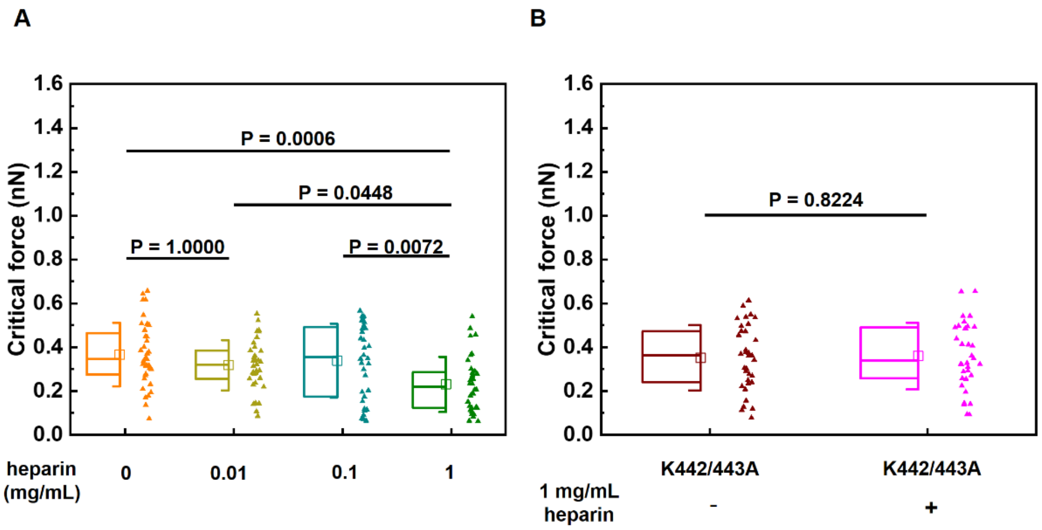

Figure S2: Critical force of HPV16 PsV after heparin treatments.
(A) Comparison of the critical force of the PsV treated by different concentrations of heparin. P values were determined by Kruskal-Wallis test with Bonferroni correction for multiple comparisons. (B) Box plot of the critical force of the K442/443A PsV incubated with or without heparin. Heparin engagement did not trigger significant critical force changes in K442/443 PsV. The significance level is 0.05. The square in the box plots indicates the mean (also printed in Table S1), the top and bottom of the box are the 25th and 75th percentiles respectively, and the whiskers represent the standard deviation. The line in the box indicates the median. Source data are provided as a Source Data file.

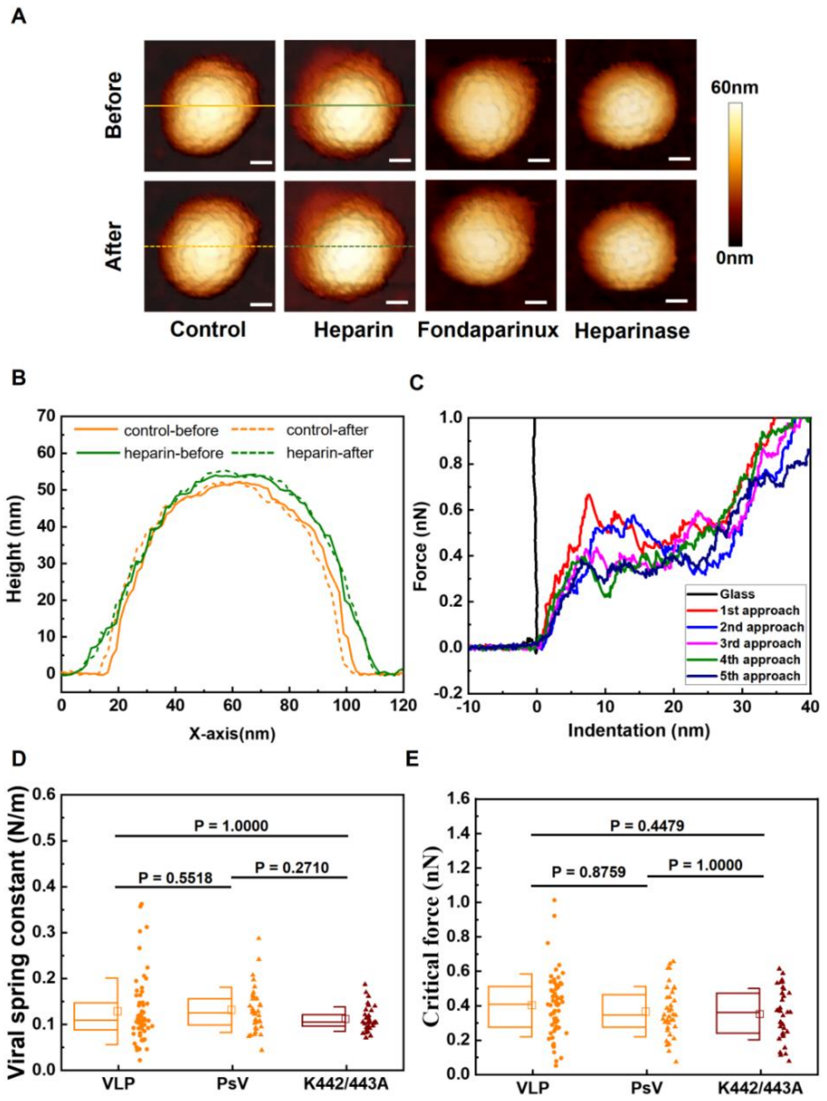

Figure S3: Deformation of HPV particles under the applied force is elastic. HPV PsV and K442/443A PsV have similar mechanical properties as HPV VLP.

(A) Representative AFM images of VLPs upon different GAG treatments before and after indentation. The scale bar is 20 nm. (B) Height profiles of untreated and heparin-treated VLPs before and after indentation. The height profiles of VLPs before and after indentation were acquired from the sections marked with solid and dashed lines in panel A). No statistically significant differences were found between the height of VLPs before and after indentation. The height of untreated VLPs (N=36) was 54 ± 2 nm (mean ± standard deviation) before indentation and 53 ± 2 nm after indentation. For heparin-treated VLPs (N=36), the height remained constant at 55 ± 2 nm before and after indentation. (C) Typical approach lines of the force-indentation curves of an untreated VLP for five successive indentations. The black curve was acquired by using an AFM tip to indent glass substrate as a reference. (D) Box plot of the viral spring constant of VLP, PsV, K442/443A PsV. No significant differences in viral spring constant were found between the three groups. The significance level is 0.05 with Bonferroni correction for multiple comparisons. (E) Box plot of the critical force of VLP, PsV and K442/443A PsV. No significant differences in critical force were found between the three groups. The significance level is 0.05 with Bonferroni correction for multiple comparisons. The square in the box plots indicates the mean (also printed in Table S1), the top and bottom of the box are the 25th and 75th percentiles respectively, and the whiskers represent the standard deviation. The line in the box indicates the median. Source data are provided as a Source Data file.

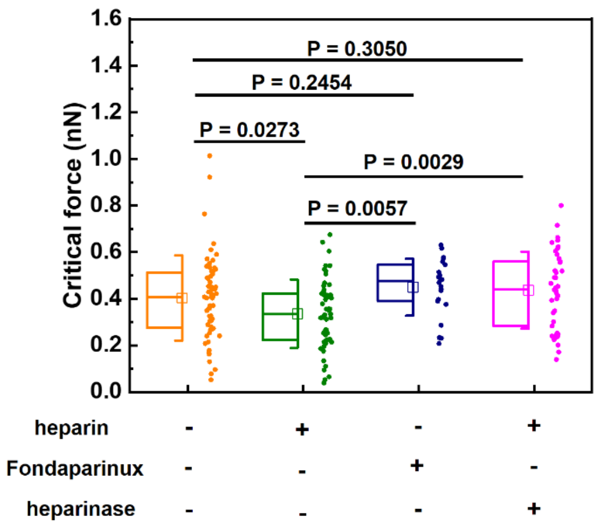

Figure S4: Critical force of HPV VLPs after different treatments.
Comparison of the critical force of the VLPs treated by heparin or Fondaparinux, and the VLPs treated by heparinase subsequent to heparin treatment. The significance level is at 0.05. The square in the box plots indicates the mean (also printed in Table S1), the top and bottom of the box are the 25th and 75th percentiles respectively, and the whiskers represent the standard deviation. The line in the box indicates the median. Source data are provided as a Source Data file.

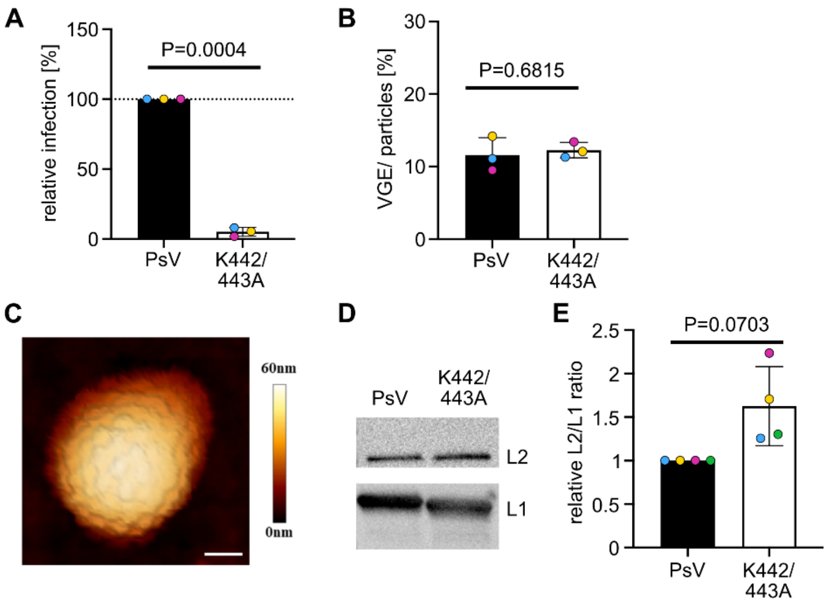

Figure S5: Characterization of K442/443A PsV preparations.
(A) PsV of either WT or mutant HPV16 were used to infect HeLa cells. GFP expression was detected 48 h p.i. by flow cytometry. Infected cells were displayed as average in % of total cells relative to the WT infection for three independent experiments ± SD. SD for the PsV control is 0 due to the normalization. (B) Viral genome equivalents (VGE) were measured by qPCR using a viral pseudogenome standard. VGE values are displayed as average in % relative to the used particle amount for three independent experiments ± SD. (C) Representative AFM image of a K442/443A PsV particle. The scale bar is 20 nm. (D) Exemplary Western blot of WT and mutant HPV16 PsV with antibodies against L2 and L1. (E) Quantification of (D). Four different K442/443A PsV preparations were assessed for the incorporation of L2 by Western Blotting as in (D). Displayed are the ratios of L2 signals over L1 signals normalized to the WT ± SD. SD for the PsV control is 0 due to the normalization. Source data are provided as a Source Data file.

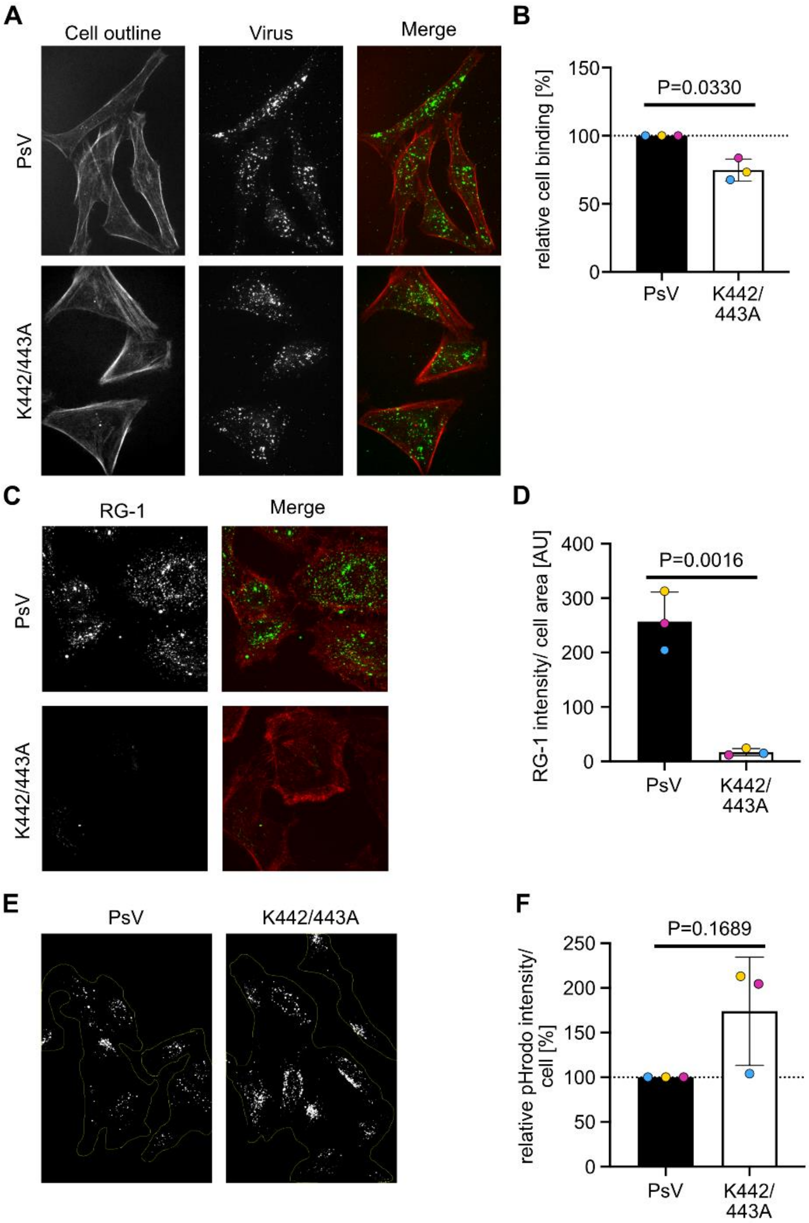

Figure S6: Analysis of K442/443A PsV during entry.
(A) PsV of either WT or mutant were fluorescently labelled and exposed to HeLa cells in the same amounts. The samples were analysed by confocal microscopy 2 h post virus addition with virus in green and actin (phalloidin staining) in red. Representative images are displayed as maximum intensity projections of confocal stacks. (B) At least 10 fields of view per replicate were analysed computationally to measure virus spots per cell area. Quantified spots were displayed as average in % relative to the WT for three independent experiments ± SD. SD for the PsV control is 0 due to the normalization. (C) PsV of either WT or mutant exposed to HaCaT cells in the same amounts. The samples were stained with an antibody against an epitope of the L2 N-terminus (RG-1 antibody) and analysed by confocal microscopy 6 h post virus addition with virus in green and actin (phalloidin staining) in red. Representative images are displayed as maximum intensity projections of confocal stacks. (D) At least 10 fields of view per replicate were analysed computationally to measure RG-1 signal intensity per cell area. Displayed is RG-1 signal intensity per cell (AU, arbitrary units) for three independent experiments ± SD. (E) PsV of either WT or mutant were covalently labelled with pHrodo, a pH sensitive dye that exhibits fluorescence under acidic conditions within endosomes, and exposed to HeLa cells in the same amounts. The samples were analysed by confocal microscopy 6 h post virus addition. Representative images are displayed as maximum

intensity projections of confocal stacks with cell outlines in yellow. (F) At least 10 fields of view per replicate were analysed computationally to measure pHrodo intensity per cell. Quantified intensities are displayed as average in % relative to the WT for three independent experiments ± SD. SD for the PsV control is 0 due to the normalization. Source data are provided as a Source Data file.

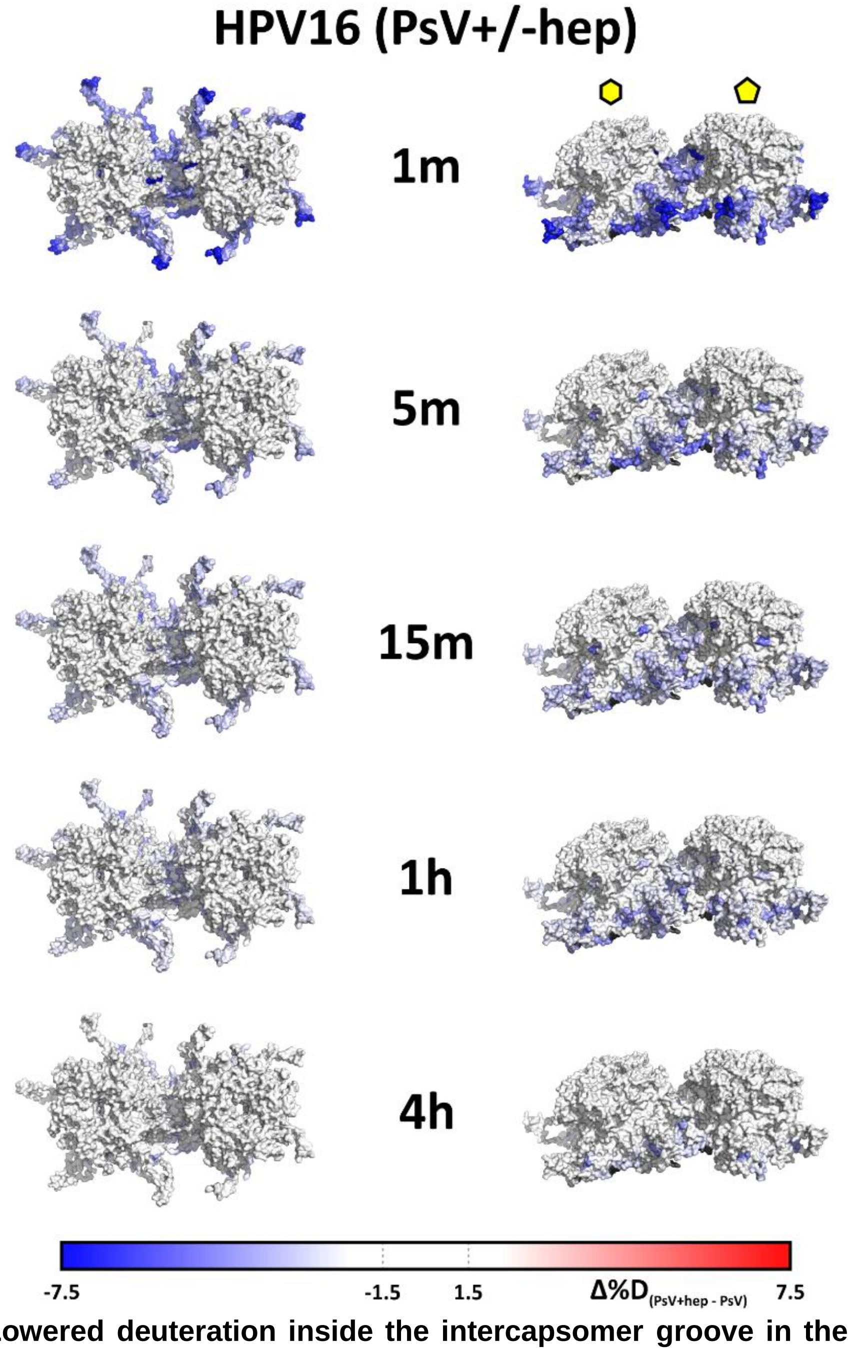

Figure S7: Lowered deuteration inside the intercapsomer groove in the presence of heparin observed over the course of the HDX.

Maximal observed differences in deuteration are visualized symmetrically on the cryo-EM HPV16 structure - hexavalent and pentavalent capsomers showed in isolation. Black regions on the structure denote missing data.

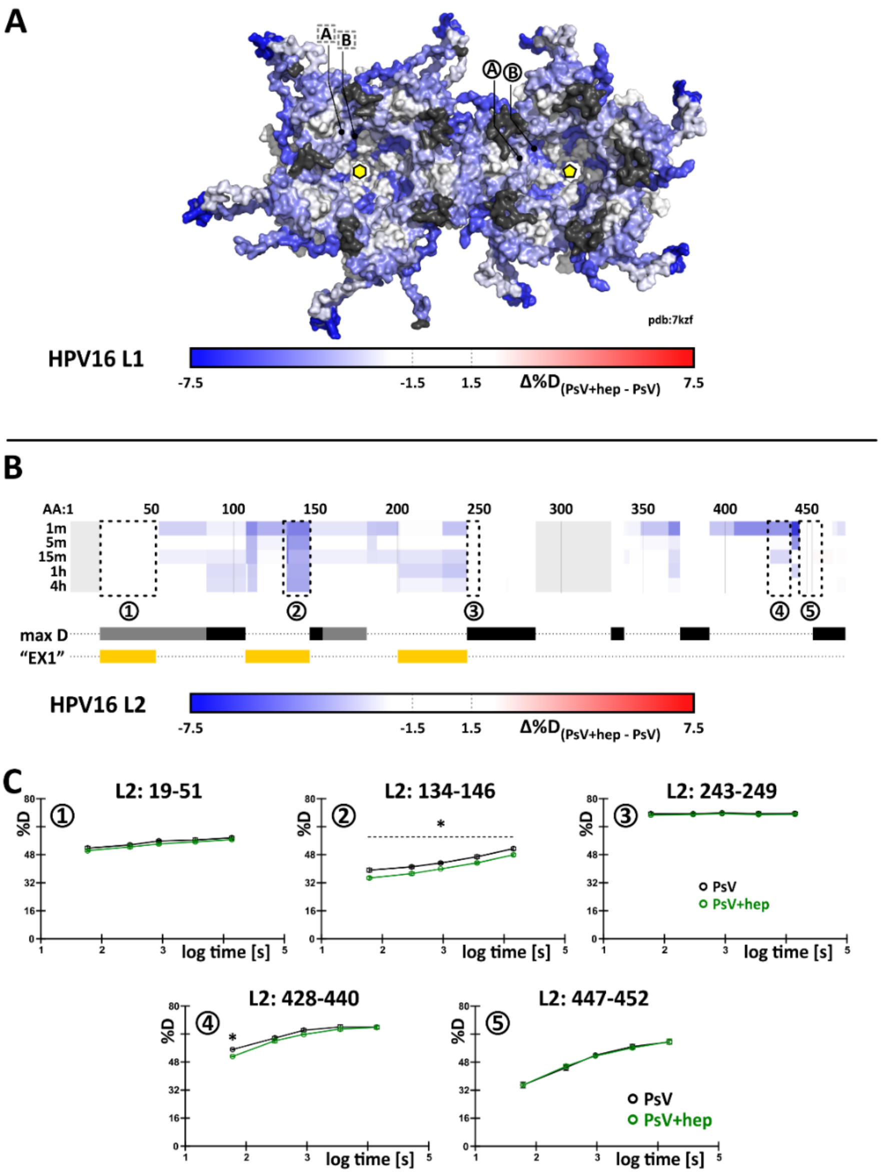

Figure S8: HDX-MS observed lowered deuteration inside the capsid in the presence of heparin.

(A) Decreased deuteration observed on the lumenal side of the capsid with heparin. Labels A and B show the region 306-327 and 247-256, respectively, marked on chain A (black circles) and chain C (dashed grey square) of the pdb structure as described in Figure 5. Black regions mark areas with lacking data. (B,C) While lacking high-resolution structural model, L2 protein also showed several regions with lowered deuteration, but additionally multiple regions with maximal deuteration achieved immediately (region 3, black bars) or almost immediately (region 1, grey bars) indicating unstructured / disordered regions. Clear bimodal EX1 HDX kinetics were detected in regions of L2 marked by yellow bars, indicating distinct conformations present in the mixture already prior to the heparin incubation and deuterium labelling. Light grey areas in (B) mark missing data. In uptake plots $N = 3$ (on deuterium labelling level). The centres in uptake plots represent mean value. Error bars show standard deviation, which is generally within the size of the point label. The significant differences (labelled with asterisk) are determined by two-tailed unpaired parametric T-test with single pooled variance ($\alpha \leqslant 0.05$, with Holm-Sidak multicomparison correction). Source data are provided as a Source Data file.

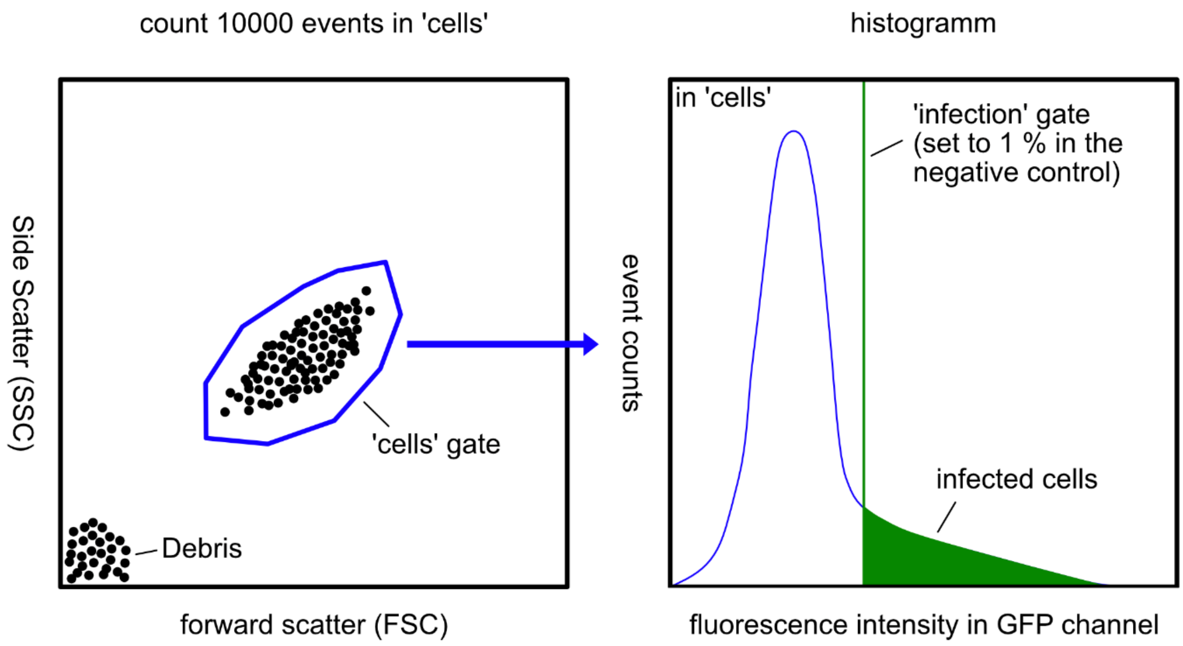

Figure S9: Gating strategy to identify GFP expressing, i.e. infected, cells by flow cytometry. Left: Viable cells were gated by forward and sideward scatter on non-infected cells excluding cell debris and single outliers. This gate was then applied to all samples (note, that forward and sideward scatter is not affected by infection), and the gated cells were then analyzed for GFP expression (488nm excitation). Within the histogram a gate was set on non-infected cells to result in 0.5-1% ‘GFP positive’ cells and this gating was applied to all samples.

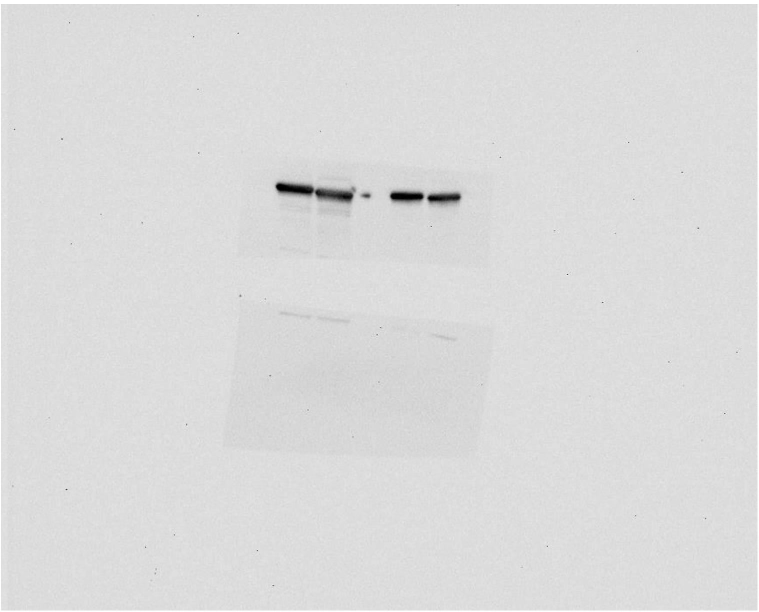

Figure S10: Full scan of the Western blot analysis of WT and mutant HPV16 PsV samples probed with antibodies against L1 and L2, corresponding to the data presented in Figure S5D. The lanes, from left to right, represent HPV16 WT, HPV16 K442/443A mutant, and two internal controls. The upper portion of the blot shows the L1 signal, while the lower portion displays the L2 signal.

Table S1: Statistics of the physical properties of HPV particles measured under the indicated experimental conditions by AFM. Errors are standard deviation

Particle Type	Experimental Conditions	Height (nm)	Viral spring constant (N/m)	Critical force (nN)
HPV16 PsV	Control (N = 36)	52 ± 2	0.13 ± 0.05	0.37 ± 0.15
	0.01 mg/mL Heparin (N = 35)	53 ± 3	0.11 ± 0.02	0.32 ± 0.11
	0.1 mg/mL Heparin (N = 34)	54 ± 2	0.10 ± 0.04	0.34 ± 0.17
	1 mg/mL Heparin (N = 36)	54 ± 2	0.09 ± 0.03	0.23 ± 0.13
K442/443A	Control (N = 34)	53 ± 1	0.11 ± 0.03	0.35 ± 0.15
	1 mg/mL Heparin (N = 34)	53 ± 1	0.12 ± 0.13	0.36 ± 0.15
HPV16 VLPs	Control (N = 60)	54 ± 2	0.13 ± 0.07	0.40 ± 0.18
	1 mg/mL Heparin (N = 55)	55 ± 2	0.09 ± 0.07	0.36 ± 0.15
	1 mg/mL Fondaparinux (N = 22)	53 ± 3	0.13 ± 0.08	0.45 ± 0.12
	Heparin + Heparinase (N = 41)	54 ± 1	0.12 ± 0.03	0.43 ± 0.16

Table S2: Summary statistics for HDX-MS experiments. For full HDX-MS data exports in a txt format according to community guidelines as well as raw LC-MS mass spectra please refer to the ZENODO link in the data availability section of the main manuscript.

Data Set	HPV16 PsV unbound	HPV16 PsV + 1 mg/ml heparin
HDX reaction details	150 mM NaCl, 4.8 mM KCl, 10 mM Na_2HPO_4, 1.8 mM KH_2PO_4, 0.9 mM $CaCl_2$, 0.5 mM $MgCl_2$, pD 7.2, 22°C	
HDX time course (min)	1, 5, 15, 60, 240	
Quench	0.25 M glycine-Cl, 100 mM TCEP, 8 M urea, quenched pH 2.5, wo/w 1 mg/ml protamine sulphate, 1.5 min at 0°C	
Protease	immobilized porcine pepsin	
# of Peptides	228 (L1) / 46 (L2)	
Sequence coverage	94% (L1) / 86% (L2)	
Average peptide length	15.7 (L1) / 17.2 (L2)	
Redundancy on covered residues	7.6 (L1) / 1.9 (L2)	
Replicates (biological or technical)	3 (technical)	
Repeatability (average standard deviation)	0.62% (L1) / 1.10% (L2)	0.50% (L1) / 0.85% (L2)
Significant differences in HDX determined by	two-tailed unpaired parametric T-test with single pooled variance ($\alpha \leqslant 0.05$, with Holm-Sidak multicomparison correction)	
